# Clinical and genomic analysis of hypermucoviscous *Klebsiella pneumoniae* isolates: Identification of new hypermucoviscosity associated genes

**DOI:** 10.3389/fcimb.2022.1063406

**Published:** 2023-01-04

**Authors:** Meiling Jin, Tianye Jia, Xiong Liu, Meitao Yang, Na Zhang, Jiali Chen, Xiaojing Yang, Shiyu Qin, Fangni Liu, Yue Tang, Yong Wang, Jinpeng Guo, Yong Chen, Boan Li, Changjun Wang

**Affiliations:** ^1^ School of Public Health, China Medical University, Shenyang, Liaoning, China; ^2^ Department of Emergency Response, Chinese People's Liberation Army Center for Disease Control and Prevention, Beijing, China; ^3^ The Clinical Laboratory, Fifth Medical Center of People's Liberation Army General Hospital, Beijing, China; ^4^ Department of Information, Chinese People’s Liberation Army Center for Disease Control and Prevention, Beijing, China; ^5^ College of Public Health, Zhengzhou University, Zhengzhou, Henan, China; ^6^ Department of Health Service, Chinese People’s Liberation Army Center for Disease Control and Prevention, Beijing, China

**Keywords:** *Klebsiella pneumonia*, hypermucoviscosity, invasive infection, risk factors, genomic analysis

## Abstract

**Introduction:**

Hypermucoviscous *Klebsiella pneumoniae* (HmKp) poses an emerging and highly pathogenic global health threat. This study aimed to investigate the clinical and genomic characteristics of HmKp isolates to better understand the virulence mechanisms of the hypermucoviscous (HMV) phenotype.

**Methods:**

From May 2018 to August 2021, 203 non-repeat *K. pneumoniae* isolates causing invasive infections were collected from a hospital in Beijing, China. Isolates were divided into HmKp (n=90, 44.3%) and non-HmKp (n=113, 55.7%) groups according to string test results.

**Results:**

Multivariate regression showed that diabetes mellitus (odds ratio [OR]=2.20, 95% confidence interval (CI): 1.20-4.05, *p*=0.010) and liver abscess (OR=2.93, CI 95%:1.29-7.03, *p*=0.012) were associated with HmKp infections. *K. pneumoniae* was highly diverse, comprising 87 sequence types (STs) and 54 serotypes. Among HmKp isolates, ST23 was the most frequent ST (25/90, 27.8%), and the most prevalent serotypes were KL2 (31/90, 34.4%) and KL1 (27/90, 30.0%). Thirteen virulence genes were located on the capsular polysaccharide synthesis region of KL1 strains. HmKp isolates were sensitive to multiple antibiotics but carried more *SHV*-type extended spectrum β-lactamase (ESBL) resistance genes (*p*<0.05), suggesting that the emergence of ESBL-mediated multidrug resistance in HmKp should be monitored carefully during treatment. Phylogenetic analysis disclosed that HmKp isolates were highly diverse. Comparative genomic analysis confirmed that the HMV phenotype is a plasmid-encoded virulence factor. Seventeen HmKp genes were highly associated with HmKp, and included *rmpAC*, 7 iron-acquisition-related genes, and *pagO*, which may promote liver abscess formation.

**Discussion:**

This investigation provides insight into the mechanisms producing the HMV phenotype.

## Introduction

*Klebsiella pneumoniae* is a Gram-negative bacillus that may subsist as a commensal bacterium of the nasopharyngeal and intestinal tracts, but may also cause life-threatening infections. A 1986 report from Taiwan of seven patients with *K. pneumoniae* pyogenic liver abscesses, bacteremia, and metastatic septic endophthalmitis (Liu et al., 1986) suggested the emergence of a hypervirulent pathotype. This newly-identified pathogen was designated as hypervirulent *K. pneumoniae* (HvKp). In 2004, differences in mucoviscosity were observed between strains that caused primary liver abscess and those that did not (Fang et al., 2004). HvKp overproduces capsular polysaccharides which result in a hypermucoviscous (HMV) phenotype (Shon et al., 2013). The HMV phenotype was defined by the formation of viscous strings of >5mm in length when a loop was used to stretch colonies on agar plates (Li et al., 2021a). HvKp infections are geographically dispersed and have emerged as a global public health concern. Throughout the past three decades, HvKp has caused epidemics in Asia, especially in South Korea, Japan, Singapore, and China. Meanwhile, sporadic cases have been reported elsewhere, and incidence rates are rising. (Yang et al., 2020; Li et al., 2021b; Wang et al., 2021). HvKp is an etiologic agent of severe and often multifocal infections in young and healthy individuals (Zhu et al., 2021).

However, diagnostic criteria of HvKp infections have still not been formally established. The genes *peg-344, iroB, iucA, _p_rmpA*, and *_p_rmpA2* all have been used to identify members of the HvKp-rich strain cohort with an accuracy of >0.95. Molecular analyses of virulence genes may be infeasible in most microbiology laboratories (Russo et al., 2018; Catalan-Najera et al., 2017). In contrast, the string test is applicable in both research and clinical settings. Recent evidence suggests that hypervirulence and hypermucoviscosity are two complementary but distinct phenotypes of *K. pneumoniae* (Russo et al., 2018). The hypermucoid phenotype of HvKp is presumably conferred by the products of *rmpA* and *rmpA2*, which are typically encoded in a large virulence plasmid. Hypermucoviscous *K. pneumoniae* (HmKp) is associated with high serum resistance and the clinical syndrome of liver abscess. Serotype KL1/KL2 isolates are generally more mucoid than those of other serotypes (Fung et al., 2002; Fang et al., 2007; Siu et al., 2012). KL1 strains are clustered uniformly within a monophyletic clade of clonal group 23, while KL2 strains are more genetically diverse (Struve et al., 2015).

Characterization of the clinical and genomic characteristics and antibiotic resistance trends of HmKp are needed to optimize clinical care, infection control efforts, epidemiological surveillance, and research studies. In this study, we investigated the clinical and genomic features of invasive HmKp isolates and tried to elucidate the pathogenic mechanisms of the HMV phenotype.

## Method and materials

### Collection of *K. pneumoniae* isolates and clinical information

We performed a retrospective study by collecting non-repeated *K. pneumoniae* isolates from patients with invasive infections at a hospital specializing in liver disease (Beijing, China) from May 2018 to August 2021. K*. pneumoniae* isolates were obtained from cultures of blood, ascites, abscess drainage, biliary tract fluid, pleural fluid, and bronchoalveolar lavage aspirate, and identified by an automated Vitek II system (bioMerieux, Balmes-Les-Grottes, France). Afterwards, whole genome sequencing-based species identification with assembled genomes was performed using Kleborate v2.2.0 (Lam et al., 2021). Clinical information was retrieved from electronic medical records, including basic demographic characteristics, underlying diseases, antimicrobial agent exposures, the site of infection, and the use of invasive devices. Hospital-acquired infections were defined as incident infections with an onset after 48 hours of admission.

### Determination of HMV phenotype

To determine the HMV phenotype, a string test was performed by touching a colony grown overnight on a blood agar plate at 37°C with a loop and pulling it up. Strains exhibiting a mucoid string with a length of ≥5 mm were defined as HmKp. Otherwise, they were classified as non-HmKp. Each string test was repeated three to five times.

### Antimicrobial susceptibility testing

Antimicrobial susceptibility testing was performed by the VITEK 2 (bioMérieux) with Vitek2 Gram Negative ID cards (AST-N335; bioMérieux) (ticarcillin, piperacillin, ceftazidime, cefoperazone, cefepime, aztreonam, imipenem, meropenem, amikacin, tobramycin, ciprofloxacin, levofloxacin, doxycycline, minocycline, trimethoprim) and broth dilution susceptibility testing (ertapenem). *K. pneumoniae* ATCC700603 was used as a quality control strain. Results were interpreted according to the recommendations of the Clinical and Laboratory Standards Institute 2020 (CLSI, 2020).

### Whole genome sequencing, multilocus sequence types and serotyping

Genomic DNA was extracted using a QIAamp DNA Mini Kit (#51306 Qiagen) following the manufacturer’s recommendations. The Illumina nova seq6000 and MGISEQ-2000 platforms were used for sequencing. De novo assembly was performed using Unicycler v0.5.0 (Wick et al., 2017). Centrifuge v1.0.4 was used to classify contigs and remove contaminating sequences (Kim et al., 2016).

Gene prediction and the annotation of assembled sequences were performed via Prokka v1.12 (Seemann, 2014). STs were determined via MLST v2.16.1 and reconfirmed using stringMLST v0.6.3 based on reads. For any strain that did not match an existing ST number, we uploaded the sequencing data to the *Klebsiella* MLST database (https://bigsdb.pasteur.fr/cgibin/bigsdb/bigsdb.pl?db=pubmlst klebsiellas eqdef&page=login) to obtain a new ST number. Capsular K serotypes were identified by whole-genome data using Kleborate v2.2.0 (Lam et al., 2021).

### Phylogenetic analysis

The collection of Prokka-annotated sequences was analyzed with Roary v3.13.0 (Page et al., 2015) to infer core and pangenomes. Maximum-likelihood phylogenetic tree analyses were generated using RAxML-NG v1.0.1 (Kozlov et al., 2019) with 1000 bootstrap replicates and the specific model selected by ModelTest-NG v0.1.7 (Darriba et al., 2020). The tree was visualized using the Figtree v1.4.4 graphical viewer (http://tree.bio.ed.ac.uk/software/figtree/) and iTOL (https://itol.embl.de.).

### Virulence genes, antimicrobial resistance genes, and plasmid analysis

The presence of resistance and virulence genes was predicted using the Comprehensive Antibiotic Resistance Database (Jia et al., 2017) and Virulence Factor Database (Chen et al., 2016), respectively, in the ABRicate v0.8.13 program. A heatmap was generated using Tbtools software (Chen et al., 2020). Platon v1.6 (Schwengers et al., 2020) was used to classify plasmid contigs and confirmed plasmids via plasmidID v1.6.5(https://github.com/BU-ISCIII/plasmidID.wiki. git).

### Comparative pangenome analysis

Pangenome analysis was performed using Roary v3.13.0 (Page et al., 2015). Rarefaction and accumulation curves were created using R version 4.2.1. Functional annotation of genes was done on RAST using the SEED subsystems approach. For RAST annotation, nucleotide files were uploaded to RAST by default features (RAST annotation scheme: RASTtk, automatically fix errors, fix frameshifts, build metabolic model, backfill gaps, turn on debug: yes, verbose level: 0).

### Statistical analysis

All data were analyzed using R version 4.2.1. The chi-square test or Fisher’s exact test were used to analyze categorical variables. Continuous variables were presented as means ± standard deviation (SD) and were compared using Student’s t-test. Normally distributed continuous variables were expressed as means ± SD and compared using Student’s t-test. Non-normally distributed continuous variables were presented as medians with interquartile ranges (IQR) and compared using the Mann–Whitney U test. Univariate and multivariable logistic regressions were performed to explore the risk factors of HmKp infection. All variables with a value of *p*<0.1 within univariate analysis were included in the following multiple logistic regression model. Stepwise regression was used to identify statistically significant predictors. *p*<0.05 was considered as statistically significant.

## Results

### Clinical characteristics of HmKp and non-HmKp isolates

**Figure 1:**
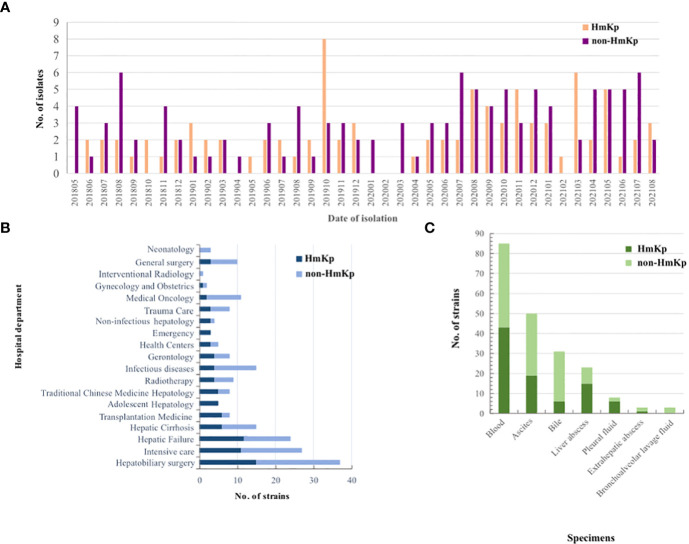
Clinical distributions of HmKp and non-HmKp isolates **(A)** Chronological distribution of HmKp and non-HmKp isolates. **(B)** Frequency of HmKp and non-HmKp isolates in hospital departments. **(C)** Distribution of HmKp and non-HmKp isolates by clinical specimen.

A total of 203 K*. pneumoniae* isolates were collected; 90 (44.3%) exhibited a positive string test and were designated as HmKp. The proportions of HmKp isolates among invasive *K. pneumoniae* isolates in 2018, 2019, 2020, and 2021 were 35.3%(12/34), 56.0% (28/50), 40.2%(27/67), and 44.2%(23/52), respectively. The χ2 test for trend indicated that the annual proportions of HmKp isolates were similar over the four-year timespan (**Figure 1A**). The four departments with the highest prevalence of HmKp were Adolescent Hepatology (5/5,100.0%), Emergency (3/3,100.0%), Uninfected Hepatology (3/4, 75.0%), and Transplantation Medicine (6/8,75.0%) (**Figure 1B**). HmKp was isolated most often from the bloodstream (43/90, 48%), followed by ascites (19/90, 21%) and liver abscesses (15/90,17%). Non-HmKp isolates were obtained most often from the bloodstream (42/113,37%), ascites (31/113,27%), and bile (25/113, 22%) (**Figure 1C**).

Demographic and clinical features of the 203 infected patients are listed in **Table 1**. The prevalence of diabetes mellitus (58.9% vs 36.3%; *p*=0.001) and liver abscess (26.7% vs 8.8%; *p*=0.001) were significantly higher in the HmKp than in the non-HmKp group. Solid malignancy (22.2% vs 41.5%; *p*=0.004) and digestive diseases (10.0% vs 27.4%; *p*=0.002) were more prevalent in the non-HmKp group. Additionally, there was a lower proportion of patients with HmKp infections undergoing drainage (32.2% vs 47.8%; *p*=0.025); *p*=0.035) compared to patients with non-HmKp infections. Fever was more common in the HmKp group than in non-HmKp group (74.4% vs 60.2%; *p*=0.032). Fewer patients were treated with penicillin in the HmKp group than in the non-HmKp group (7.8% vs 19.5%; *p*=0.018).

**Table 1:** Demographic and clinical characteristics of 203 patients with hypermucoviscous *Klebsiella pneumoniae* (HmKp) or non-hypermucoviscous *K. pneumoniae* infection.

Characteristics	HmKp (n = 90) n (%)	non-HmKp (n = 113) n (%)	*p*-value
Age (years)*	55 (48, 63)	58 (49, 67)	0.159
Male	65 (72.2)	85 (75.2)	0.629
Hospital-acquired infection	53 (58.9)	78 (69.0)	0.134
Underlying diseases			
Solid malignancy	20 (22.2)	47 (41.5)	0.004
Liver cirrhosis	52 (57.8)	72 (63.7)	0.389
Digestive diseases	9 (10.0)	31 (27.4)	0.002
Diabetes mellitus	53 (58.9)	41 (36.3)	0.001
Chronic kidney disease	22 (24.4)	41 (36.3)	0.070
Hypertension	11(12.2%)	23(20.4%)	0.123
Pulmonary disease	16 (17.8)	28 (24.8)	0.229
Clinical presentation			
Bacteremia	5 (5.6%)	4 (3.5%)	0.726
Peritonitis	21 (23.3)	32 (28.3)	0.422
Liver abscess	24 (26.7)	10 (8.8)	0.001
Extrahepatic abscess	3 (3.3)	2 (1.8)	00.796
Use of invasive devices			
Drainage tube	29 (32.2)	54 (47.8)	0.025
Intravenous/Venous catheter	22 (24.4)	38 (33.6)	0.154
Urinary catheter	11 (12.2)	15 (13.2)	0.824
Gastrostomy tube	9 (10.0)	16 (14.2)	0.370
Bone marrow puncture	0 (0)	6 (5.3)	0.072
Endotracheal intubation	0 (0)	5 (4.4)	0.118
Surgical procedures	7 (7.8)	15 (13.3)	0.211
Length of hospital stay (days)	22.3 ± 16.0	29.5 ± 21.2	0.006
Symptoms/Signs			
Fever	67 (74.4)	68 (60.2)	0.032
Nausea	4 (4.4)	12 (10.6)	0.105
Weakness	32 (35.6%)	49 (43.4%)	0.259
Cough	8 (8.9)	5 (4.4)	0.197
Antibiotic exposures			
Penicillins	7 (7.8)	22 (19.5)	0.018
Carbapenems	13 (14.4)	25 (22.1)	0.163
Cephalosporins	25 (27.8)	28 (24.8)	0.629

*Age data are presented as mean (standard deviation) or median, (interquartile range).

Bold values indicate p < 0.05.

### Risk factors for invasive HmKp infections

Subsequent to the above analysis, all factors from univariate analysis with a *p*<0.1 were included in the multivariate model. In the multivariate analysis, diabetes mellitus (odds ratio [OR]=2.20, 95% confidence interval [CI]=1.20-4.05; *p*=0.010) and liver abscess (OR=2.93, 95%CI=1.29-7.03; *p*=0.012) were associated with increased odds of HmKp infection. Solid malignancy (OR=0.45, 95%CI=0.23-0.85; *p*=0.016) and digestive diseases (OR=0.38,95%CI=0.15-0.86; *p*=0.025) were associated with markedly decreased odds of HmKp infection (**Table 2**).

**Table 2:** Clinical risk factors for HmKp infection identified by *logistic* regression.

Variables	Multivariate analysis	
	OR (95%CI)	p-value
Liver abscess	2.93 (1.29-7.03)	0.012
Diabetes mellitus	2.20 (1.20-4.05)	0.010
Solid malignancy	0.45 (0.23-0.85)	0.016
Digestive diseases	0.38 (0.15-0.86)	0.025

OR, odds ratio; CI, confidence interval.

### Multilocus sequence typing and serotyping

**Figure 2:**
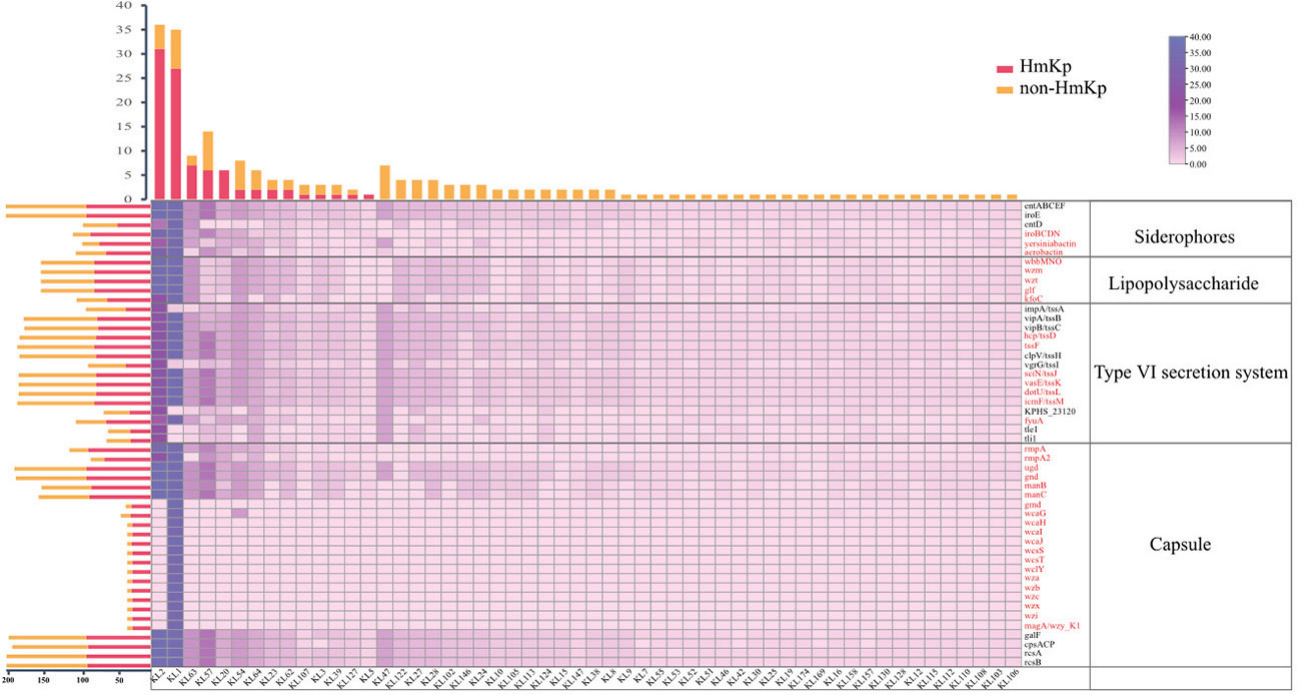
Distributions of siderophores, lipopolysaccharide, type VI secretion system, and capsule-related genes among serotypes. The bar graph (top) represents the numbers of isolates by serotype. The bar graph (left) represents the numbers of virulence genes in two groups. Groups are indicated by different colors. The numbers of virulence genes are depicted as a heatmap. The red font represents genes that were significantly more prevalent in the HmKp group than in non-HmKp group.

*K. pneumoniae* isolates were highly diverse, comprising 87 STs and 54 serotypes. Eight of the STs (ST6105, ST6106, ST6107, ST6108, ST6109, ST6110, ST6111, and ST6112) were first identified in this study. The most frequent STs in the HmKp group were ST23(25/90, 27.8%) and ST65 (15/90, 16.7%). In the non-HmKp group, the most frequent ST was ST11 (12/113, 10.6%) (**Figure S1**). The most frequent serotypes in the HmKp group were KL2 (31/90, 34.4%) and KL1(27/90, 30.0%), which accounted for >50% of isolates (**Figure 2**). Interestingly, most KL1 strains belonged to ST23 (31/35, 88.6%), and 25 of KL1-ST23 strains were HmKp.

### Profiling virulence-associated genes

A total of 126 virulence genes were recorded, with KL1 isolates having the highest repertoire (120/126, 95.2%). Thirteen virulence genes located on the capsular polysaccharide synthesis (*cps*) region were identified only in the KL1 serotype. The prevalence of 79 genes was statistically higher in HmKp; these included genes encoding colibactin; allantoin utilization; aerobactin, yersiniabactin, salmochelin, and lipopolysaccharide (LPS) synthesis; the type VI secretion system (*tssD*, *tssF*, *tssJ*, *tssK*, *tssL*, *tssM*, *fyuA*); and the gene cluster for capsule production (*rmpA*, *rmpA2*, *ugd*, *gnd*, *manB, manC*, *gmd*, *wcaG, wcaH, wcaI, wcaJ*, *wcsS, wcsT*, *wclY*, *wza, wzb, wzc, wzi, wzx*, *magA*) (*p*<0.05) (**Figure 2**). In addition, 112 HvKp isolates were defined by various combinations of *peg-344*, *iroB*, *iucA*, *_p_rmpA*, or *_p_rmpA2*, and included 88 HmKp. In the HmKp group, only two isolates were not HvKp.

### Analysis of antibiotic resistance phenotypes and genotypes

HmKp isolates were generally more susceptible than non-HmKp isolates to antimicrobial agents that included meropenem, imipenem, aztreonam, cefepime, ceftazidime, ciprofloxacin, levofloxacin, piperacillin, tobramycin, doxycycline, minocycline, and ticarcillin (*p*<0.05) (**Table S2**). Only one of 14 carbapenem-resistant isolates was an HmKp (ST11-KL64). Notably, the prevalence rates of *SHV*-type ESBL genes (*SHV-11*, *SHV-190*, *SHV-207*) were higher in the HmKp group than in the non-HmKp group (*p*<0.05) (**Figure S2**).

### Phylogenetic analysis based on core genes

**Figure 3:**
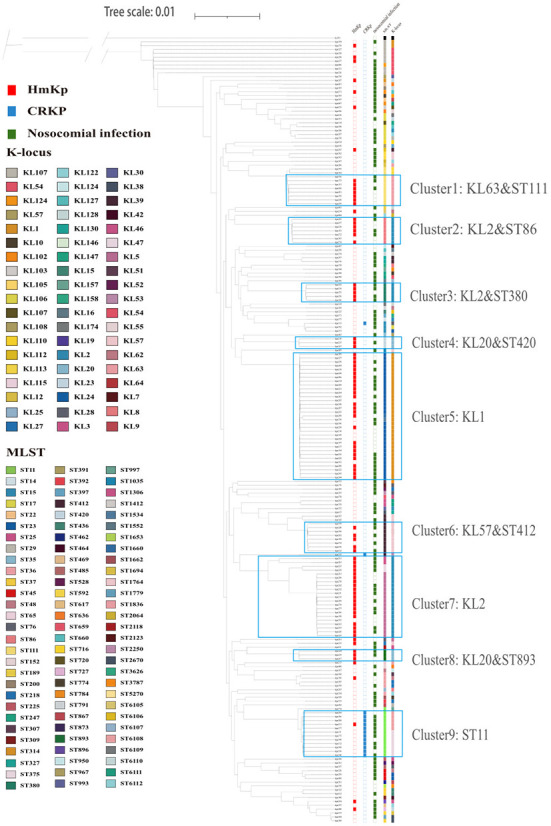
Core-gene phylogeny of 203 K*. pneumoniae* isolates. Isolates are annotated with datasets of HmKp, carbapenem-resistant isolates (CRKP), hospital-acquired infection, sequence types(ST) and capsular locus(KL) from left to right. Different STs and serotypes are designated by color, and the several ST clusters are marked. The most dominant clusters of HmKp and CRKP isolates are marked as: Cluster 1: KL63-ST111; Cluster 2: KL2-ST86; Cluster 3: KL2-ST380; Cluster 4: KL20-ST420; Cluster 5: KL1; Cluster 6: KL57-ST412; Cluster 7: KL2; Cluster 8: KL20-ST893; Cluster 9: ST11.

The core genome-based phylogenetic tree is shown in **Figure 3**. *Klebsiella variicola subsp. Variicola*, kv291 collected from the same hospital during the study period was used as an outgroup to root the tree. A cluster was defined as a large branch with the same serotype and sequence type. Most isolates had clearly distinct phenotypes in the cluster, such as HMV and carbapenem resistance. According to these criteria, we identified 9 clusters. Cluster 5 was the largest, and was comprised entirely of KL1 strains. KL2 isolates clustered in different sub-branches with different STs (Cluster 2: KL2 & ST86; Cluster 3: KL2 & ST380; Cluster 7: KL2). HmKp strains were scattered in different clusters whereas carbapenem-resistant (CPKP) strains were grouped closely in Cluster 9 (ST11).

To evaluate the phylogenetic relationships of the 203 K*. pneumoniae* isolates in public genomes, we selected 116 K*. pneumoniae* strains from PATRIC to construct a phylogenetic tree together with the 203 isolates in our study using the method described above. We downloaded complete gene sequences of 116 *K. pneumoniae* isolates causing invasive infections worldwide from 2018-2021. Our 203 isolates did not cluster together but were scattered within the clusters of public genomes. HmKp isolates were dispersed throughout the phylogenetic tree, consistent with our phylogenetic analysis (**Figure S3**).

### Comparative genomic analysis

**Figure 4:**
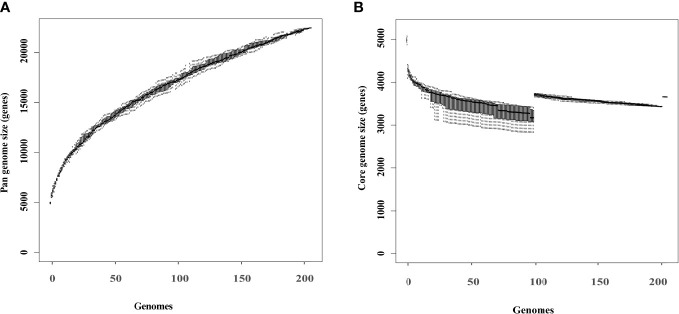
Rarefaction curves of the core and pangenome of 203 *K pneumoniae* isolates. Box plots show the estimated size of the pangenome **(A)** and core genome **(B)**.

The pangenome of 203 K*. pneumoniae* isolates consisted of 22435 genes, of which 3652 (16.3%) belonged to the core genome, and 18783 (83.7%) were accessory (**Figure 4**). Rarefaction and accumulation curves were indicative of an open pangenome with a well-defined core, and suggested that additional genomes were unlikely to impact the size of the core genome much further. We conducted additional analysis to distinguish genes between the HmKp and non-HmKp groups. We applied the accuracy rate as an indicator, and found that 17 of 22435 genes were highly associated with HmKp (**Table 3**). When a particular gene was used as a biomarker for HmKp, accuracy rate was calculated by dividing the sum of the number of isolates carrying the gene in the HmKp group and the number of isolates without the gene in non-HmKp group divided by the total number of isolates (Ye et al., 2016).

**Table 3:** Identification of 17 genes associated with hypermucoviscosity.

Gene/ORF names	The prevalence of genes in HmKp group	The prevalence of genes in non-HmKp group	Location of contig in kpn191	Putative annotation
NCTC5050_00289	85/90	22/113	EOMPELDC_04883	Mobile element protein
UUU_03070	86/90	26/113	EOMPELDC_04884	Putative SAM-dependent methyltransferases
*rmpA*	85/90	22/113	EOMPELDC_04885	Mucoid phenotype regulator
*rmpC*	85/90	24/113	EOMPELDC_04886	Mucoid phenotype regulator
*pagO*	85/90	24/113	EOMPELDC_04887	DMT family transporter
NCTC5046_05205	85/90	24/113	EOMPELDC_04888	Hypothetical protein
*iroN*	85/90	24/113	EOMPELDC_04889	Siderophore receptor
*iroD*	83/90	22/113	EOMPELDC_04890	Putative ferric enterochelin esterase
*iroC*	86/90	24/113	EOMPELDC_04891	Salmochelin/enterobactin export ABC transporter
*iroB*	85/90	24/113	EOMPELDC_04892	Putative glucosyltransferase
*parB*	84/90	23/113	EOMPELDC_04893	N-terminal domain-containing protein
*ibrA*	84/90	23/113	EOMPELDC_04894	Phosphoadenosinephosphosulfate reductase
uncharacterized	84/90	23/113	EOMPELDC_04895	Hypothetical protein
NCTC5046_05192	84/90	23/113	EOMPELDC_04896	IS3 family transposase
*fecI*	84/90	23/113	EOMPELDC_04897	Putative RNA polymerase sigma factor
*fecR*	84/90	23/113	EOMPELDC_04898	Transmembrane signal transducer for ferric citrate transport
*fecA*	84/90	22/113	EOMPELDC_04899	Ferric citrate outer membrane transporter

Identical genes were identified manually on UniProt and BLAST.

The 17 predicted HmKp-associated genes were located on a 21889 bp DNA fragment carried by the pK2044 plasmid and also detected on a 191041 bp pM186-like plasmid from kpn191. Among the 17 genes, rmpA is a known HmKp associated gene, and 7 genes (*iroB, iroC, iroD, iroN, fecA, fecI, fecR*) are related to iron-acquisition. The liver abscess-causing *K. pneumoniae* (LAKP)-associated gene *pagO* was also encoded in this fragment.

### Functional Comparative Analyses of HmKp

**Figure 5:**
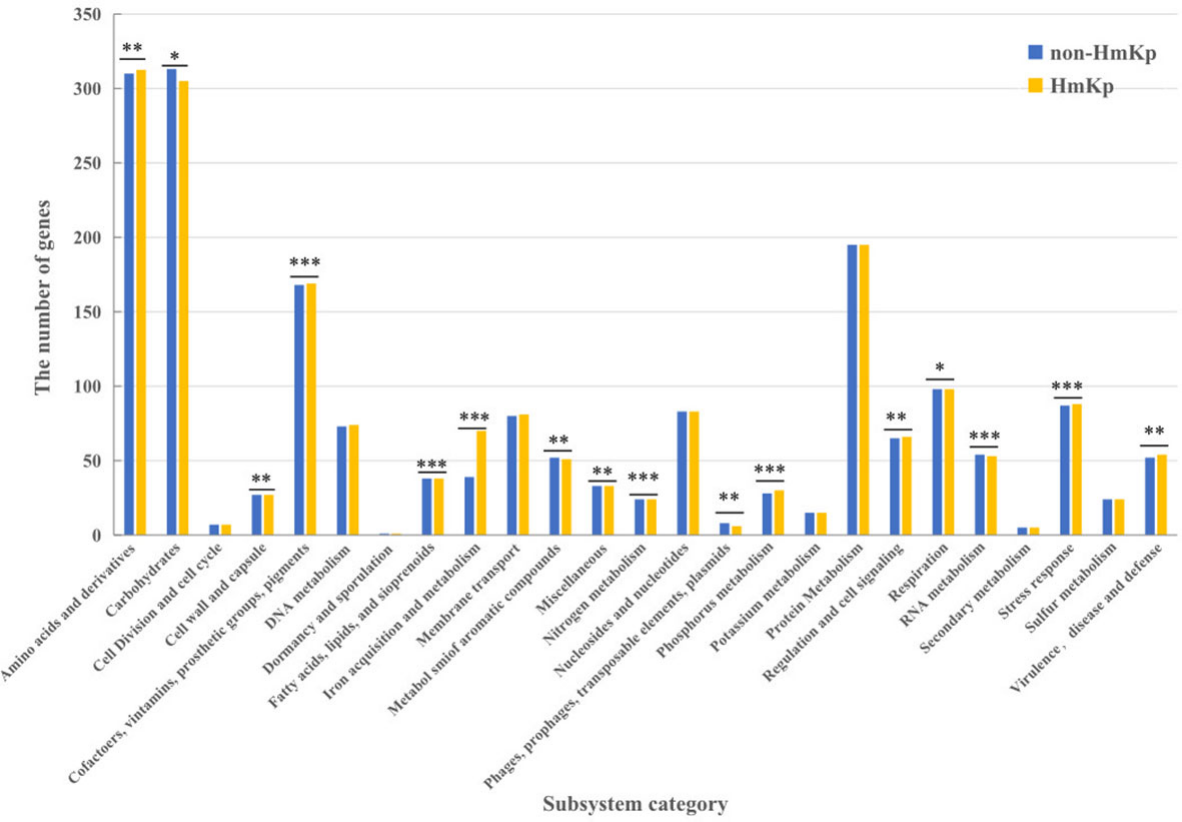
Classifications of gene models into functional categories for HmKp and non-HmKp. Gene classifications were performed with RAST functional annotations and the SEED subsystem database .*p < 0.05, **p < 0.01, ***p < 0.001; n.s., not significant..

Classification of genes into discrete functional units provides valuable insight into how resources are allocated to each function. We functionally annotated the *K. pneumoniae* genome using RAST, resulting in the classification of genes into 25 subsystems. To assess HmKp related functions, median numbers of genes of each group were compared at the RAST subsystem level. As shown in **Figure 5**, sixteen categories showed significant difference between HmKp and non-HmKp isolates.

Most notably, the number of genes in the “Iron acquisition and metabolism” category was significantly higher in HmKp than in non-HmKp isolates. The numbers of genes in the “Virulence, Disease and Defense”, “Regulation and Cell signaling,” “Stress Response,” “Cofactors, Vitamins, Prosthetic Groups, Pigments,” “Fatty Acids, Lipids, and Isoprenoids,” “Nitrogen Metabolism,” “ Amino Acids and Derivatives,” and “Phosphorus Metabolism” categories were significantly greater in HmKp compared to non-HmKp isolates. In addition, non-HmKp isolates had significantly more members of genes in 6 other categories (“Cell Wall and Capsule,” “Phages, Prophages, Transposable elements, Plasmids,” “RNA Metabolism,” “Respiration,” “Miscellaneous” “Metabolism of Aromatic Compounds,” and “Carbohydrates”) than HmKp strains (**Figure 5**).

## Discussion

HvKp has received increasing worldwide attention due to its high pathogenicity (Sanchez-Lopez et al., 2019). Compared with HvKp, HmKp is easily identified but poorly studied. We analyzed 203 invasive *K. pneumoniae* isolates to reveal a few important characteristics by dividing them into HmKp and non-HmKp groups. We found 17 HmKp associated genes that provided a foundation for subsequent studies on the mechanisms by which the HMV phenotype arises.

HmKp strains are being isolated from Chinese patients with increasing frequency. Although HmKp prevalence did not show a temporal trend in our study, its epidemiology and virulence warrant further study. An association between HmKp and liver abscess was shown as early as 2004 (Fang et al., 2004). Diabetes mellitus has been considered a significant risk factor of HmKp infections. The bactericidal capacity of diabetic neutrophil extracellular traps against HmKp may be impaired, which partially explains the susceptibility of patients with diabetes to HmKp infections (Jin et al., 2020). Similarly, we found that liver abscess and diabetes were associated with increased odds of HmKp infection.

We observed that HmKp exhibited antibiotic-susceptible phenotypes but carried more *SHV*-type ESBL genes than non-HmKp isolates, linking the HMV phenotype to unexpressed *SHV* and *TEM*-type ESBLs. Therefore, monitoring of antimicrobial resistance is required throughout an entire treatment course due to the potential of antibiotic-induced expression of resistance genes. Our observation is consistent with earlier associations of serum resistance with *SHV* and *TEM*-positive strains (Hennequin and Robin, 2016). Studies showed that serum-resistant isolates were associated with ESBL-producing strains (*SHV* and *TEM*-types) than non-ESBL-producing strains (Hennequin and Robin, 2016). The property of serum resistance depends on the capsule synthesis that protects the bacteria from phagocytosis (Sahly et al., 2004). And the increased capsule production contributes to the mucoid phenotype.

Increased capsule production contributes to the mucoid phenotype. KL1 and KL2 strains are frequently associated with HmKp and are more virulent than strains of other serotypes (Yu et al., 2008). The number of genes in the “Virulence, Disease and Defense” category was significantly higher in HmKp than in non-HmKp isolates. KL1 isolates were clustered uniformly within a monophyletic clade of clonal group 23, while KL2 strains were more genetically diverse. Furthermore, capsular polysaccharide-mediated virulence is related to the high resistance of K1 and K2 capsules to monocytic phagocytosis (Zhang et al., 2016). Thirteen virulence genes promoting capsule production are located on the capsular polysaccharide gene cluster of KL1 strains. This may explain the frequent HMV phenotype among KL1 strains.

However, overproduction of capsular polysaccharides was not the only mechanism. We were surprised to find that the number of genes in the “Cell Wall and Capsule” category was significantly higher in non-HmKp than in HmKp isolates. The mucoid phenotype synthesis protein *rmpD* is essential for the HMV phenotype but does not impact the capsule (Walker et al., 2020). In addition to overproduction of capsular polysaccharides, the HMV phenotype may be related to many other factors. We found that HmKp isolates carry more genes associated with colibactin, LPS, siderophores, and allantoin utilization. LPS mutations may affect capsule retention or biosynthesis (Dorman et al., 2018). As the outermost subunit of LPS, the O antigen may defend against complement-mediated killing. A reported association between the mucoid phenotype and aerobactin production needs to be substantiated by additional studies (Yu et al., 2007).

The analysis of the phylogenetic tree disclosed a degree of genetic diversity among our HmKp isolates. Carbapenem-resistant strains were primarily clustered in ST11 clones, but HmKp isolates were phylogenetically dispersed among STs and serotypes. This suggests that the evolutionary signal is insufficient to explain this distribution. Alternatively, this phenomenon may be explained by horizontal gene transfer. This assumption was next elucidated by comparative pangenome analysis. Seventeen HmKp-associated genes were often plasmid-encoded, suggesting that the HMV phenotype was probably acquired by plasmid transfer. As early as 1989, a virulence-encoding plasmid pKP200 in *K. pneumoniae* was demonstrated to encode the mucoid phenotype (Nassif et al., 1989)

Among the 17 genes, *rmpA*, which encodes a positive regulator of capsular polysaccharide biosynthesis, is closely associated with the hypervirulent phenotype (Yu et al., 2006). These results clearly demonstrate the feasibility and accuracy of the method to predict the HmKp-associated genes. *rmpC*, a gene with a predicted LuxR-type DNA binding domain, is necessary for full *manC* expression. Overexpression leads to elevated *manC* expression but loss of the HMV phenotype. However, cooperativity between *rmpA*, *rmpC*, and *_p_rmpAC* led to increased mucoviscosity and normal *manC-gfp* expression. *rmpC* is not the key gene for regulating the HMV phenotype, but co-regulates with *rmpA*. The protein encoded by *pagO* may be similar to a product of the *Yersinia* virulence plasmid product (Gunn et al., 1998). A murine model suggests that *pagO* in LA-Kp may be required for liver abscess formation (Tu et al., 2009). Furthermore, Ye et al. (2016) identified 30 LAKP-associated genes, of which 21 were newly-discovered. A substantial and significant overlap was found between HmKp-associated and LAKP-associated genes. The close relationship between HmKp and liver abscess was also confirmed by logistic regression. Ye et al. suggested that these gene expressions may promote both the HMV phenotype and liver abscess formation.

The *iroB*, *iroC*, *iroD* and *iroN* genes encode the synthesis, excretion, and uptake of salmochelin. In *Escherichia coli*, *fecA*, *fecI* and *fecR* are ferric citrate transport genes (Enz et al., 2000). Iron is a critical element required for essential metabolic processes of both bacteria and their hosts (Shon et al., 2013). Our functional enrichment analysis revealed that the number of genes in the “Iron acquisition and metabolism” category was significantly higher in HmKp than in non-HmKp isolates. However, few studies have investigated the regulatory mechanism of capsular polysaccharides and iron acquisition. Lin et al. (2011) demonstrated that Fur regulates capsular polysaccharides biosynthesis in a Fe (II)-dependent manner. The relationship between iron acquisition and the HMV phenotype warrants further research.

Our study has several limitations. Firstly, this study was conducted in a single hospital. HmKp infections in other hospitals may have different clinical characteristics and distinct genetic structures. Secondly, genomic characteristics of invasive *K. pneumoniae* isolates could not be adequately assessed due to the limited number of cases. Thirdly, HmKp-associated genes were predicted by only using comparative genomic analysis. Consequently, biological studies of isogenic mutants are necessary.

In conclusion, this study showed that diabetes mellitus and liver abscess were associated with higher risks of HmKp infection. HmKp isolates were genetically diverse; the HMV phenotype may be a plasmid-encoded virulence factor. Furthermore, we identified 17 genes highly related to HmKp with an accuracy of over 85%. These genes included a mucoid phenotype regulator (*rmpAC*), a liver abscess-associated gene (*pagO*), and iron acquisition-related genes (*iroBCDN*, *fecAIR*). Our results may facilitate further studies of the mechanism of HMV phenotype.

## Data availability statement

The datasets presented in this study can be found in online repositories. The names of the repository/repositories and accession number(s) can be found in the article/**Supplementary Material**.

## Ethics statement

The procedures for obtaining the patient data were reviewed and approved by the Ethics Committee of Fifth Medical Center of PLA General Hospital (approval ID #KY-2021-10-19-1). The requirement for written informed consent from the participants was waived.

## Author contributions

MJ, TJ, and XL contributed equally in this study. MJ, MY, NZ, FL and XY isolated bacteria and performed the laboratory measurements. YC, CW and BL made substantial contributions to conception and design. YT, YW, JG and TJ revised the manuscript critically for important intellectual content. XL, JC and SQ participated in experimental design and data analysis. MJ drafted the manuscript. All authors read and approved the final manuscript. All authors contributed to the article and approved the submitted version.
