# Supplementary material for: Clinical and genomic analysis of hypermucoviscous Klebsiella pneumoniae isolates: Identification of new hypermucoviscosity associated genes
Source: Front Cell Infect Microbiol. 2023 Jan 4;12:1063406. doi: 10.3389/fcimb.2022.1063406 (PMC9846069; doi:10.3389/fcimb.2022.1063406)
Supplement: Supplementary file 1 [file Table_1.docx]

**Supplementary material**

**Table S1.** Comparison of resistance between two groups.

|  | HmKp (n=90)  n (%) | non-HmKp (n=113)  n (%) | *P*-value |
| --- | --- | --- | --- |
| Ertapenem | 1 (1.1) | 5 (4.4) | 0.333 |
| **Meropenem** | **1 (1.1)** | **13 (11.5)** | **0.004** |
| **Imipenem** | **1 (1.1)** | **13 (11.5)** | **0.004** |
| Amikacin | 4 (4.4) | 10 (8.8) | 0.219 |
| **Aztreonam** | **3 (3.3)** | **29 (25.7)** | **<0.001** |
| **Cefepime** | **3 (3.3)** | **25 (22.1)** | **<0.001** |
| **Ceftazidime** | **1 (1.1)** | **25 (22.1)** | **<0.001** |
| Trimethoprim | 90 (100) | 108 (95.6) | 0.118 |
| **Ciprofloxacin** | **6 (6.7)** | **42 (37.2)** | **<0.001** |
| **Levofloxacin** | **9 (10.0)** | **55 (48.7)** | **<0.001** |
| **Piperacillin** | **1 (1.1)** | **17 (15.0)** | **0.001** |
| **Tobramycin** | **1 (1.1)** | **15 (13.3)** | **0.001** |
| **Doxycycline** | **8 (8.9)** | **72 (63.7)** | **<0.001** |
| **Minocycline** | **3 (3.3)** | **23 (20.4)** | **<0.001** |
| **Ticarcillin** | **1 (1.1)** | **25 (22.1)** | **<0.001** |

Bold values indicate P<0.05

**Figure S1.** Presence of siderophores, lipopolysaccharide (LPS), type VI secretion system and capsule-related genes in *K. pneumoniae* isolates according to STs. The bar graph represents the numbers of isolates by STs. Groups are indicated by colors. The numbers of virulence genes are depicted as a heatmap. Red font represents genes more prevalent in the HmKp than in the non-HmKp group.

**Figure S2.** Distribution of genes encoding extended-spectrumβ-lactamases (ESBLs) (bla_CTX-M_, bla_SHV_, bla_TEM_) among 203 *K. pneumoniae* isolates. **p*<0.05, ***p*<0.01, ****p*<0.001; n.s., not significant.

**Figure S3.** Core gene phylogeny of *Klesiella pneumoniae* isolates comprising 203 from this study and 117 from public databases. Isolates are annotated in datasets of collection year, source, and country from outer to inner circles (1-3), respectively. Isolates from public databases are in green shades. Isolates from this study are shown in yellow and HmKp are in red font.
