# Supplementary figures and images for: Clinical and genomic analysis of hypermucoviscous Klebsiella pneumoniae isolates: Identification of new hypermucoviscosity associated genes
Source: Front Cell Infect Microbiol. 2023 Jan 4;12:1063406. doi: 10.3389/fcimb.2022.1063406 (PMC9846069; doi:10.3389/fcimb.2022.1063406)

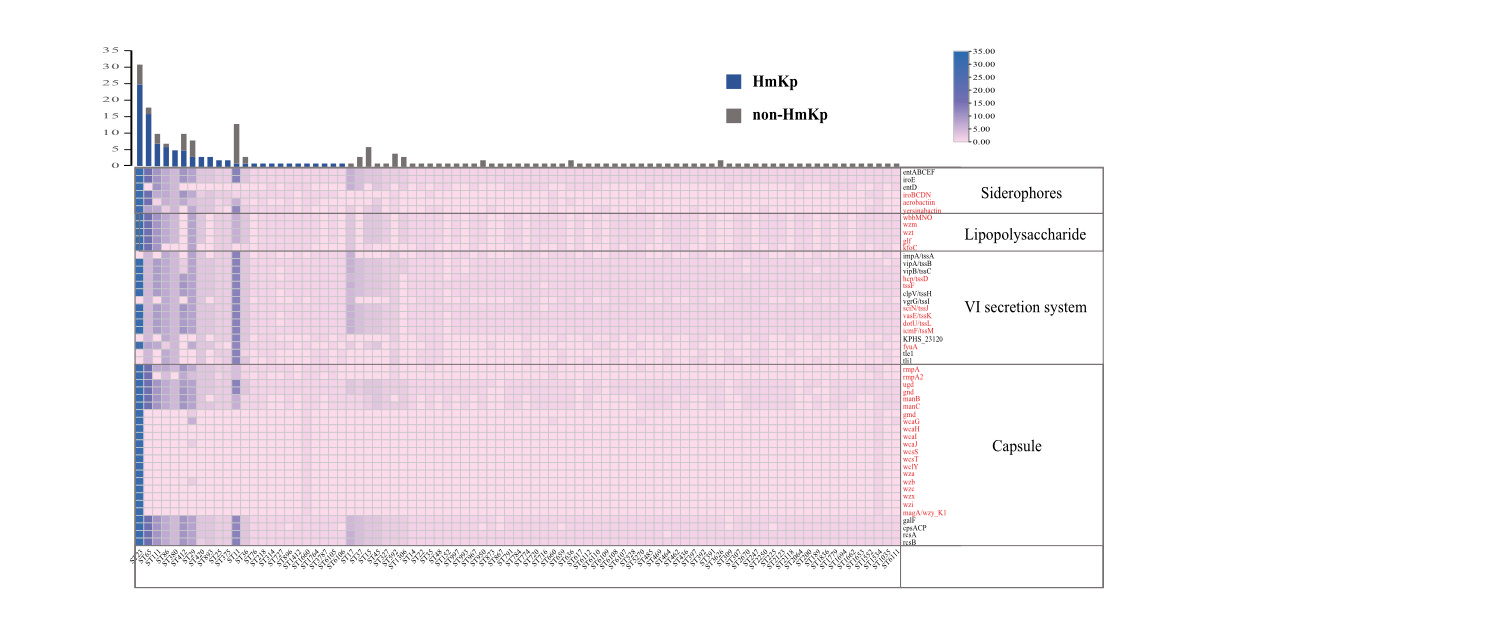

Supplement: Supplementary file 2 [file Image_1.tif]

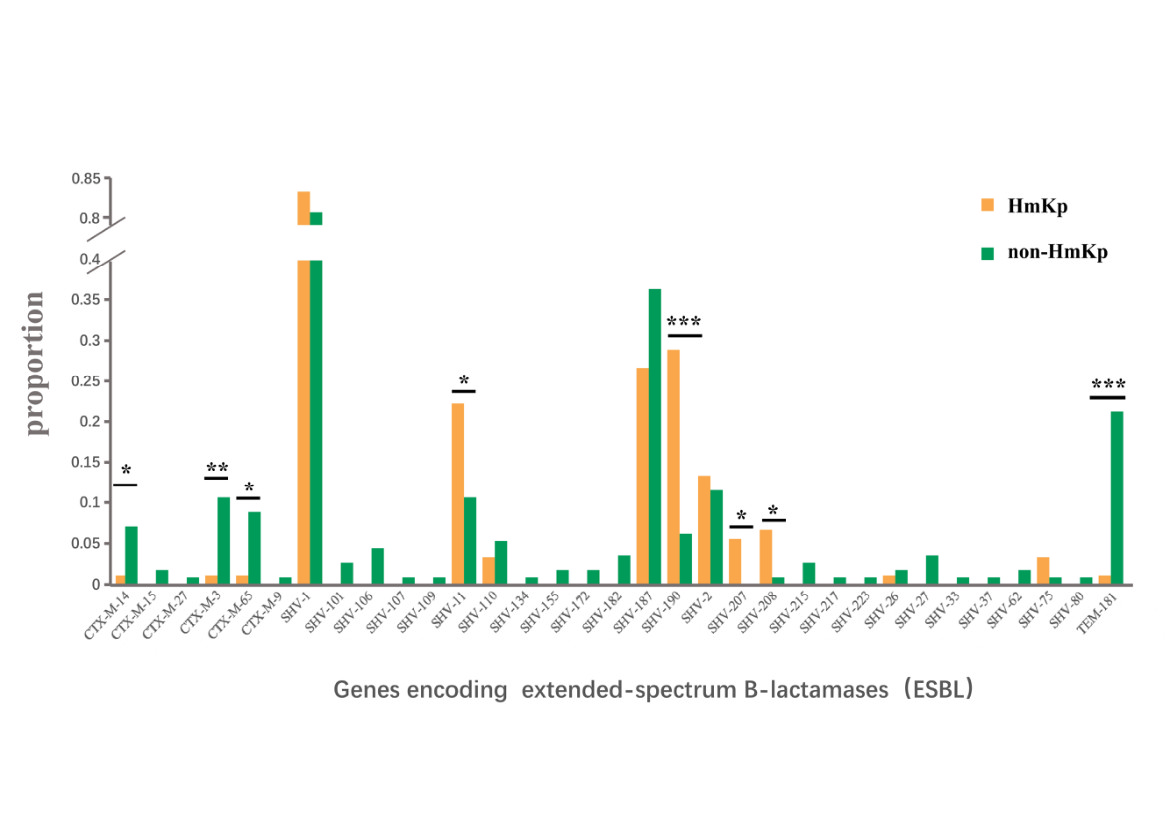

Supplement: Supplementary file 3 [file Image_2.tif]

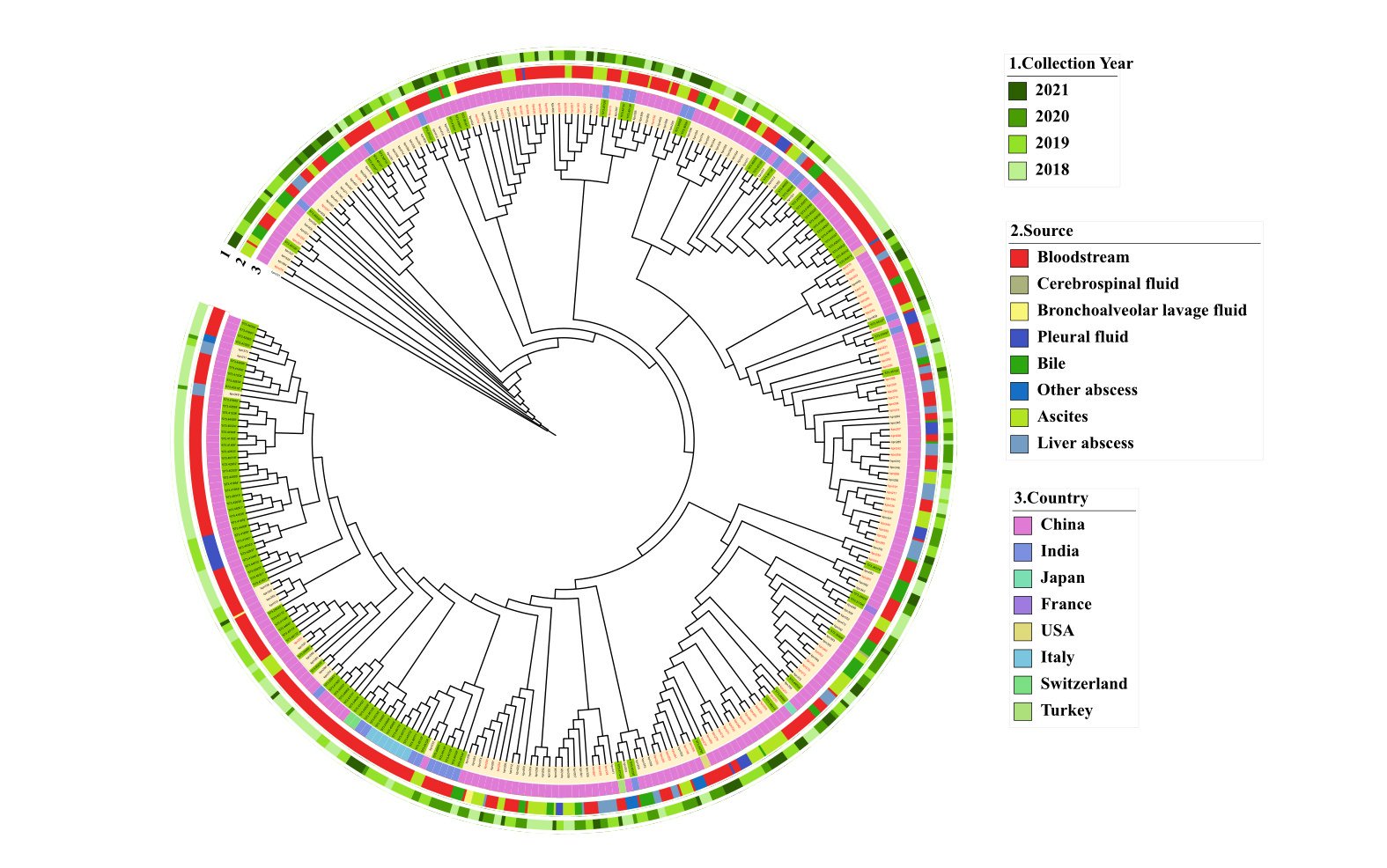

Supplement: Supplementary file 4 [file Image_3.tif]
